# Tuning the photocatalytic/electrocatalytic properties of MoS_2_/MoSe_2_ heterostructures by varying the weight ratios for enhanced wastewater treatment and hydrogen production†

**DOI:** 10.1039/d1ra01760h

**Published:** 2021-06-28

**Authors:** Divya Monga, Soumen Basu

**Affiliations:** School of Chemistry and Biochemistry, Affiliate Faculty—TIET-Virginia Tech Center of Excellence in Emerging Materials, Thapar Institute of Engineering and Technology Patiala-147004 India soumen.basu@thapar.edu

## Abstract

Two-dimensional (2D) heterojunctions with layered structures give high flexibility in varying their photocatalytic/electrocatalytic properties. Herein, 2D/2D heterostructures of MoS_2_/MoSe_2_ with different weight-ratios (1 : 1, 1 : 3, and 3 : 1) have been prepared by a simple one-step microwave-assisted technique. The characterization studies confirm formation of crystalline MoS_2_/MoSe_2_ nanoparticles with a high surface area (60 m^2^ g^−1^) and porous structure. The high synergistic-effect (1.73) and narrow bandgap (∼1.89 eV) of the composites result in enhanced photo-degradation efficiency towards methylene blue dye (94%) and fipronil pesticide (80%) with high rate constants (0.33 min^−1^ and 0.016 min^−1^ respectively) under visible light. The effect of pH, catalyst dose, and illumination area on photodegradation has been optimized. Photodegradation of real-industrial wastewater shows 65% COD and 51.5% TOC removal. Trapping experiments confirm that holes are mainly responsible for degradation. The composites were highly reusable showing 75% degradation after 5-cycles. MoS_2_/MoSe_2_ composites show excellent electrochemical water-splitting efficacy through hydrogen-evolution-reaction (HER) exhibiting a stable high current density of −19.4 mA cm^−2^ after 2500 cyclic-voltammetry (CV) cycles. The CV-plots reveal high capacitance activity (*C*_dl_ value ∼607 μF cm^−2^) with a great % capacitance retention (>90%). The as-prepared 2D/2D-catalysts are highly active in sunlight and beneficial for long-time physico-chemical wastewater treatment. Moreover, the electrochemical studies confirm that these composites are potential materials for HER activity and energy-storage applications.

## Abstract 2

The 2D/2D-MoS_2_/MoSe_2_ catalysts with good photocatalytic/electrocatalytic properties can be potential materials for wastewater treatment and hydrogen production.

## Graphical abstract

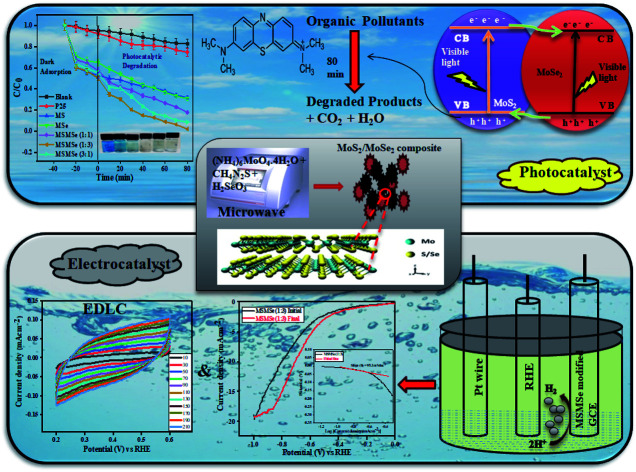

## 1. Introduction

Conserving water and energy is crucial not only for our current needs but also for the future of mankind. The world is achieving new heights with the development of science, technology, society, and mankind but at the cost of our limited resources.^1^ As we are aware, water is getting contaminated gradually^2^ and there is an enormous requirement for clean water, so it is very important to pay heed to the treatment of wastewater.^3^ Also, conventional energy sources are depleting rapidly, and their burning causes environmental hazards like global warming and climate change.^4^ Thereby, researchers are putting an emphasis on the development of alternative efficient energy sources (like hydrogen energy) which are eco-friendly, everlasting, abundant, renewable, and can replace the existing energy sources potentially.^5^ Photocatalysis is a fascinating approach to attenuate the problem of environmental pollution especially water pollution^6^ although many other techniques have also been studied. It has the potential to degrade the organic compounds to almost completely degraded products like water and carbon dioxide or some less complex and non-toxic compounds.^7^ A trend of hydrogen production from water splitting either through photocatalysis or electrocatalysis was observed with the increasing technology. The production of hydrogen as an energy source through hydrogen evolution reaction (HER) by electrochemical water splitting is one of the best ways for the green, renewable and non-toxic hydrogen production.^8^

The two-dimensional (2D) materials formed by the combination of different 2D semiconductive materials are the potential candidates for various applications like catalysis, energy storage and electronics *etc.* by tuning its electronic properties. Transition metal dichalcogenides (TMDC, MX_2_ where M = Mo, W and X = S, Se, and Te) is the class of 2D materials with a wide range of promising application prospects.^9^ These metal sulfides, selenides, *etc.* possess a special layered structure that acts as a support to anchor semiconductor nanoparticles and to provide more active sites to function and reduce electron mobility.^10^ MoS_2_ is found to be a catalyst with a wide-spectrum response because of its narrow bandgap (≈1.8 eV). It has high natural abundance, high chemical stability, low cost, high catalytic performance, and more number of surface active sites.^11^ However, the catalytic performance of MoS_2_ is unsatisfying due to the high recombination efficiency of generated charge carriers. This restraint can be overwhelmed by the construction of semiconductor heterojunctions which may effectively improve the separation efficiency of charge carriers and provide more exposed active sites which further enhance the photo/electrocatalytic activity of materials.^12^

MoSe_2_ is an interesting narrow-band-gap semiconductor that has a lamellar crystal structure, whose basic crystal unit is built of Se–Mo–Se sandwich layers similar to MoS_2_. From theoretical band-structure calculations and photoelectron spectroscopy analyses, it is indicated that the energy gap of MoSe_2_ (1.4–1.7 eV) matches the solar spectrum very well.^13^ It is also reported that MoSe_2_ possesses very high anti-photo-corrosion stability because the optical transitions of MoSe_2_ are between nonbonding metal ‘d’ states. Both of these outstanding features of MoSe_2_ greatly benefit its potential use in catalytic related applications, such as photo-electrochemical solar cells, contamination remediating agents, and hydrogen production from water splitting.^14^ Recently, MoS_2_ and MoSe_2_ nanocomposites have been obtained by various physical and chemical strategies including chemical vapor deposition (CVD), electro-deposition, colloidal synthesis, sonochemical synthesis, and solvothermal conversions. Most of the methods are very complicated and need to be carried out at high temperatures.^15^ The vertically aligned MoS_2_–MoSe_2_ nanosheets were synthesized by Yang *et al.* the liquid-phase sonication method. Yang prepared a few-layered MoS_2_ from the exfoliation of bulk MoS_2_ by sonication and the MoSe_2_ was synthesized by solvothermal technique taking exfoliated MoS_2_ as a template.^16^ The hydrothermal route was used by Li *et al.* for the fabrication of MoS_2_/MoSe_2_ heterostructures. First MoS_2_ was prepared by the hydrothermal reaction of Na_2_MoO_4_ with CH_4_N_2_S and the MoSe_2_ was prepared by the selenization of as-prepared MoS_2_ nanosheets.^17^ Ren *et al.* fabricated MoSe_2_@MoS_2_ composites by a two-step hydrothermal method. The Na_2_MoO_4_ and hydrazine hydrate with Se powder was used to prepare MoSe_2_ nanosheets, and these nanosheets were added to Na_2_MoO_4_ and l-cysteine for hydrothermal treatment to prepare MoSe_2_@MoS_2_ core–shell composites.^18^ However, the poor solubility and lower density of selenium often lead to less contact between reactants which results in an incomplete reaction. Besides, hydrazine hydrate presents higher toxicity which is not suitable for extensive use.^19^ The microwave-assisted synthesis presents various advantages like low-temperature synthesis, short reaction time, and formation of well-exposed active sites by instantaneous local heating.^20^

In this work, an attempt has been made to prepare a series of MoS_2_/MoSe_2_ nanocomposites with various weight ratios (1 : 1, 1 : 3, and 3 : 1) from a facile microwave technique. The composites were prepared *in situ* to ensure the formation of high-quality heterostructures. Their photocatalytic performance was investigated by the degradation of MB dye, fipronil pesticide, and real industrial wastewater under irradiation of different light sources (UV, visible, and sunlight). The weight ratio effect of MoS_2_ and MoSe_2_ on the photocatalytic activity was studied which shows that MoS_2_/MoSe_2_ (1 : 3) composite shows the best catalytic activity for pollutants degradation. The scavenger study has been done to determine the degradation mechanism. The effect of illumination area, pH, and catalyst dose on the photoactivity of the catalyst was also studied. The reusability efficiency of the catalyst was also optimized by several degradation studies and catalyst characterization after degradation. The electrochemical water splitting performance and stability of the synthesized composites was analyzed in the acidic medium. The electric double layer (EDLC, *C*_dl_) storage capacity and high capacitance retention rate for 2500 CV cycles show its promising application as electrode material for supercapacitors.

## 2. Material and methods

### 2.1 Synthesis of pure MoS_2_ and MoSe_2_ and their composites

**Scheme 1:**
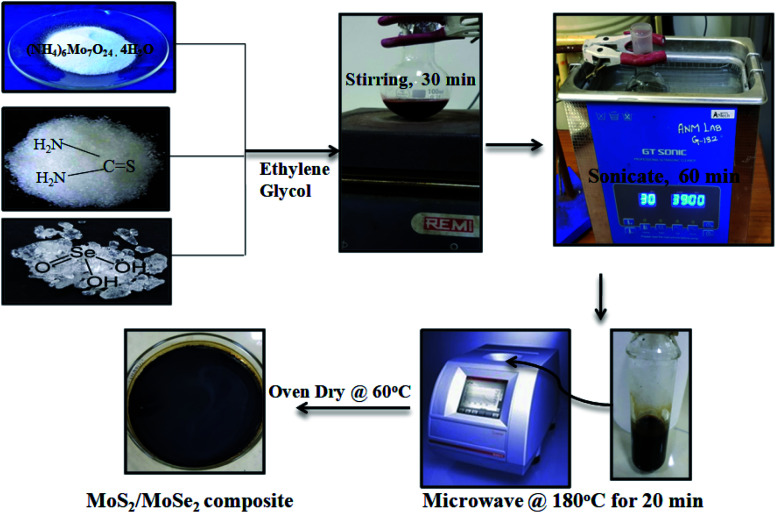
Schematic illustration for the synthesis of MoS_2_/MoSe_2_ composites.

Various weight ratios of MoS_2_/MoSe_2_ composites were prepared by a fast one-step microwave method. Firstly, the required amount of ammonium molybdate tetrahydrate, thiourea, and selenous acid was dissolved in 30 mL ethylene glycol with stirring for 30 min. After that, the mixture was sonicated for an hour to ensure the complete dispersion of the precursors. The MoS_2_/MoSe_2_ composites were prepared by heating this mixture in a microwave synthesizer (Anton Paar Monowave 300) at 180 °C for 20 min. The black-brown precipitates were washed several times with distilled water and then dried in an oven at 60 °C to obtain powder catalysts which are denoted as MSMSe (1 : 1), MSMSe (3 : 1), and MSMSe (1 : 3) according to the weight ratios of MoS_2_ and MoSe_2_ respectively. Pure MoS_2_ and MoSe_2_ were also prepared at similar conditions by using precursors of only one compound.^21^ A diagrammatic representation of synthesis procedure for MoS_2_/MoSe_2_ photocatalysts is shown in Scheme 1.

## 3. Results and discussions

### 3.1 Material characterization

#### 3.1.1 XPS

**Fig. 1:**
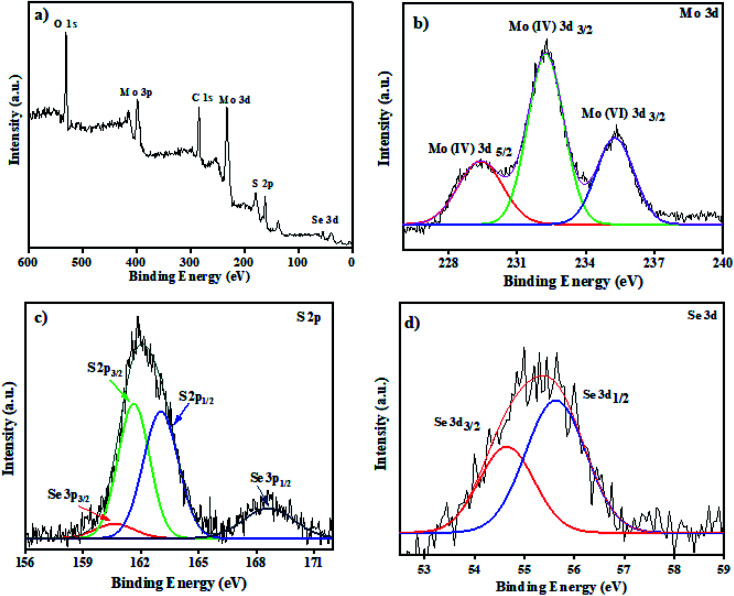
XPS analysis (a) survey spectra of MoS_2_/MoSe_2_ (1 : 1) nanocomposite, (b), (c), and (d) are the high-resolution spectra of Mo 3d, S 2p and Se 3d, respectively.

The chemical state of MoS_2_/MoSe_2_ nanocomposites was explored by using XPS analysis. The survey spectra in Fig. 1a show the presence of Mo, S, and Se elements in the composites. In Fig. 1b there are three peaks, centered around 229.2 eV and 232.2 eV, which belongs to Mo(iv) 3d_5/2_ and Mo(iv) 3d_3/2_ correspondingly indicating the presence of Mo^4+^ in the heterostructure. The higher energy peak detected at 235.3 eV arises from the 6+ oxidation state of Mo, probably due to the formation of MoO_3_. The peak appeared due to oxidation of sample exposed to air during XPS analysis.^22^ The peaks in Fig. 1c could be fitted to S 2p_1/2_ and S 2p_3/2_ at 161.3 and 162.1 eV, respectively. The binding energies of Se 3d_3/2_ and Se 3d_1/2_ in Fig. 1d is located at 54.5 eV and 55.4 eV for Se 3p_3/2_ and Se 3p_1/2_ at 160.5 eV and 168 eV which attributes to the Se^2−^ in the prepared composites.^16^

#### 3.1.2 EDS and color mapping

Energy-dispersive X-ray spectroscopy results (Fig. SI 1a†) reveal the coexistence and homogeneous distribution of Mo, S, and Se elements in MoS_2_/MoSe_2_ composite, indicating a uniform distribution of MoS_2_ and MoSe_2_ in the heterostructures. The elemental color mapping of MSMSe (1 : 3) nanocomposite with corresponding FESEM image shows the elements (Mo, S and Se) present in the sample (Fig. SI 1b–e†).

#### 3.1.3 UV-visible DRS

**Fig. 2:**
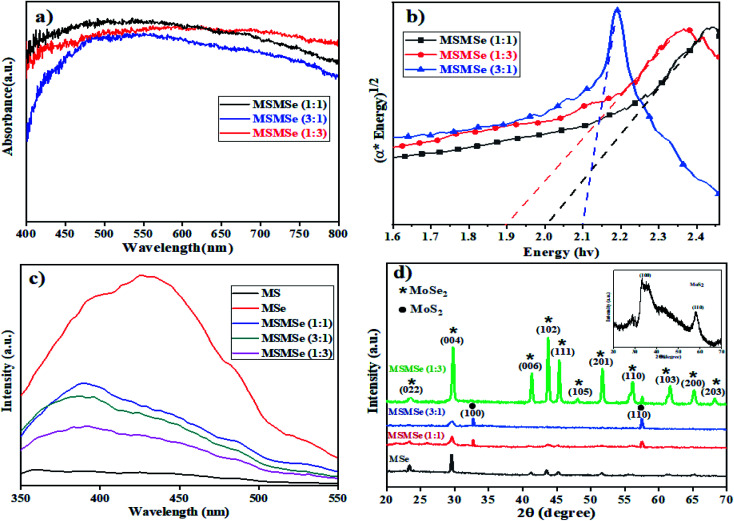
Plots of (a) DRS absorption spectra, (b) bandgap analysis, (c) photoluminescence, and (d) XRD pattern of the as-prepared MSMSe photocatalysts.

Fig. 2(a) and (b) represents the UV-visible DRS spectra of as-prepared MoS_2_/MoSe_2_ composites. Both MoS_2_ and MoSe_2_ exhibited a wide light absorption range in visible light (Fig. SI 2†). The broad absorption starting around 450 nm is attributed to the direct transition from the deep valence band to the conduction band of MoS_2_ and MoSe_2_.^23^ It was observed that pure MoS_2_ and MoSe_2_ have a bandgap of 2.21 eV and 1.66 eV respectively (inset of Fig. SI 2†), which demonstrates, both are active in the visible region of light. The bandgap of MSMSe (1 : 1) and MSMSe (3 : 1) composites are 1.99 eV and 2.1 eV respectively, signifying the good absorption of visible light. The narrow bandgap of MSMSe (1 : 3) composite (∼1.89 eV) compared with other composites might be responsible for the high photocatalytic activity of this composite due to better light absorption. The value of the conduction band (CB) and valence band (VB) was also determined by the eqn (1) and (2).^24^

1 EVB = *X* − *E*_e_ + 0.5*E*_g_

2 ECB = EVB − *E*_g_

Here EVB and ECB represent the energy of the valence band and conduction band respectively and *E*_g_ is the bandgap of the sample and *E*_e_ is the free electron energy estimated on the hydrogen scale which has a fixed value of 4.5 eV. The *X* in the equation stands for the total electronegativity of the compound.^25^ Putting the values in the above equations the value of EVB and ECB for MoS_2_ were found to be 1.25 eV and −0.94 eV respectively. Similarly, the valence band and conduction band energy of MoSe_2_ was calculated to be 1.46 eV and −0.20 eV. Due to the matching energy levels between MoS_2_ and MoSe_2_, the heterojunction formed between these two materials could assist the movement and separation of photogenerated charge carriers which enhance the photocatalytic activity of the composite.

#### 3.1.4 Photoluminescence study

The catalytic response of a material is highly dependent on the recombination rate of charges. The low photoluminescence (PL) intensity implies adequate charge separation viability and a low recombination rate of electrons and holes.^26^ In the current study, the PL-spectra of as-synthesized materials were evaluated as shown in Fig. 2c. The broad PL spectra of samples consist of two overlapping Gaussian peaks: one is centered at ∼420 nm while the other one at ∼490 nm (Fig. 2c). The lower energy PL emission can be ascribed to the transition between quantized energy levels and the other peak is might be due to defect state mediated transition.^27,28^ The PL intensity of all the composites prepared was lower than the pure MoSe_2_ which indicates that the photocatalytic activity of the composites is higher as compared to individual components because of the higher separation efficiency of the photo-induced charges in the composites. However, better separation of the charges in composites is the result of the formation of heterojunctions between MoS_2_ and MoSe_2_ which results in better electron transport. Moreover, the PL intensity of MSMSe (1 : 3) is the lowest among the composites, it consequences the highest catalytic efficacy.

#### 3.1.5 XRD

The XRD patterns of MoS_2_/MoSe_2_ composites are shown in Fig. 2d. In the composites and pure MoSe_2,_ the diffraction peaks of both MoSe_2_ and Se^29^ are present at 23.3°, 29.8°, 41.2°, 43.7°, 45.1°, 48°, 51.6°, 56°, 61.6°, 65.1°, 68.2° which represent (022), (004), (006), (102), (111), (105), (201), (110), (103), (200) and (203) (*hkl*) planes respectively (JCPDS file no 77-1715) and intensity of these peaks increases as the amount of MoSe_2_ in the composite increases.^30,31^ The diffraction peaks of MoS_2_ in the composite are present at 32.6° and 57.4° which corresponds to (100) and (110) planes of MoS_2_ (JCPDS 37-1492). The XRD pattern of pure MoS_2_ having diffraction peaks at 32.9° and 58° is shown in the inset of Fig. 2d. Both MoS_2_ and MoSe_2_ are present in hexagonal crystal systems and 2H phase in the catalyst.^32,33^ The diffraction peak of MoSe_2_ at 29.8° gets broaden with a decrease in intensity as the amount of MoS_2_ increases in the composite (MSMSe (3 : 1)). As observed from the spectra that the composites have sharp peaks implying the high crystalline nature of the composites. But compared with the pure MoS_2_ and MoSe_2_ the peaks in the composite are slightly shifted and this type of trend implies close contact and higher interaction between MoS_2_ and MoSe_2_ in the composites.^34^

#### 3.1.6 Surface area studies

To analyze the surface area of the as-prepared nanocomposites, specific surface area analysis was carried out. Fig. SI3 (a)† represents the type IV Langmuir adsorption isotherm with sharp branches of H1 hysteresis loop of all the composites along with bare MoS_2_ and MoSe_2_, which validate the mesoporous nature of the as-prepared samples. To figure out the pore size distribution of the samples, the BJH method (Fig. SI 3b†) was followed. From Table 1, we can observe that the surface area and pore volume of the pure MoS_2_ is quite high. The surface area and the pore volume of the composites are higher as compared to pure MoSe_2_. However, the surface area increases form MSMSe (1 : 1) < MSMSe (3 : 1) < MSMSe (1 : 3) which is directly responsible for the better photo/electrocatalytic efficiency of MSMSe (1 : 3) composite due to better adsorption of pollutants and more catalytically active surface area for HER.

**Table 1:** Surface area and pore size distribution analysis of the as-prepared samples

Samples	Specific surface area (m^2^ g^−1^)	Mean pore diameter (nm)	Mesopore volume (cm^3^ g^−1^)	Total pore volume (cm^3^ g^−1^)
MS	73	22.7	0.682	0.701
MSe	38	15.82	0.603	0.621
MSMSe (1 : 1)	44	16.23	0.612	0.639
MSMSe (1 : 3)	60	20.4	0.671	0.688
MSMSe (3 : 1)	50	18.02	0.615	0.664

#### 3.1.7 FESEM

**Fig. 3:**
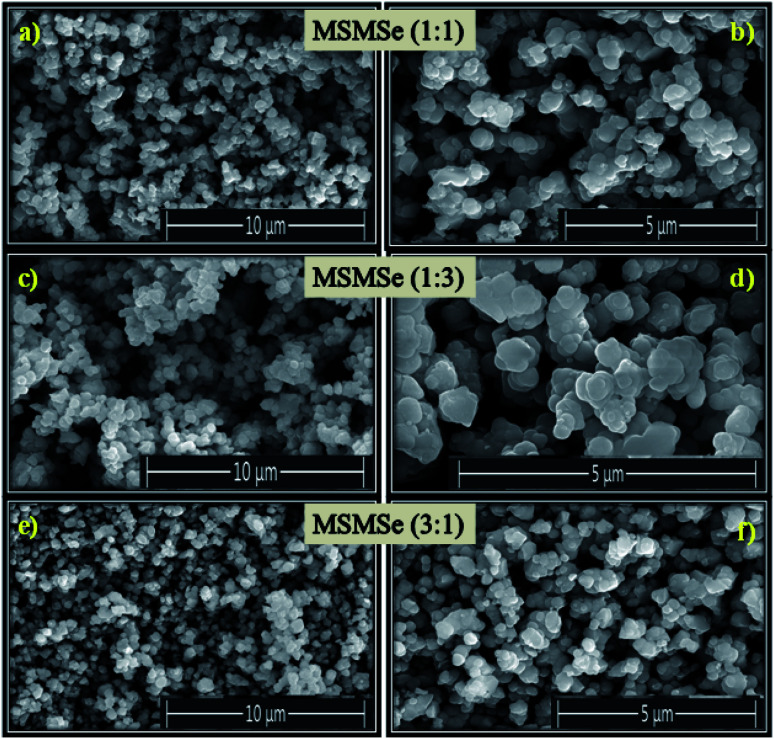
FESEM images of (a and b) MSMSe (1 : 1), (c and d) MSMSe (1 : 3) and (e and f) MSMSe (3 : 1) catalysts.

The FESEM pictures of the as-prepared composites are shown in Fig. 3(a–f). These images reveal that all the prepared composites consist of small and flat disk-like particles. FESEM pictures depict that there is an irregularity on the surface of the composites. It is difficult to differentiate the surface morphology of different composites from these FESEM images. The high-resolution image of MSMSe (1 : 3) composite (Fig. 3d) indicates that there are irregular plate/disk-like structures which may lead to the increased surface area of the catalyst, and further helps in the photo/electrocatalytic activity of the catalyst.

#### 3.1.8 TEM

**Fig. 4:**
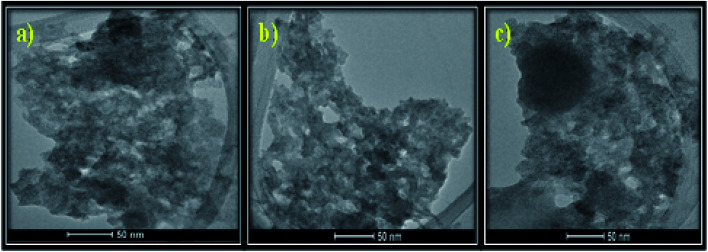
TEM images of (a) MSMSe (1 : 1), (b) MSMSe (3 : 1) and (c) MSMSe (1 : 3) nanocomposites.

Fig. 4(a–c) represents the TEM images of the as-prepared MSMSe composites which shows the presence of small aggregated spherical particles. From the TEM images, it is difficult to differentiate between different composites. The close contact of MoS_2_ and MoSe_2_ in the composites can be observed from the image. Moreover, the small pores present in the samples validate that the composite possesses a high surface area which is responsible for the high catalytic activity of the composites.

### 3.2 Photocatalytic studies

#### 3.2.1 Photodegradation of dye and pesticide

**Fig. 5:**
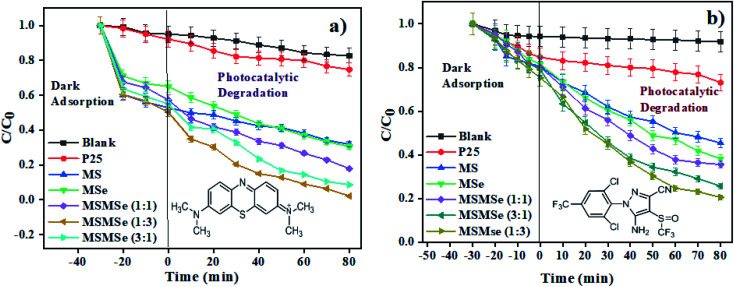
Kinetic analysis showing photodegradation of (a) methylene blue dye and (b) fipronil pesticide.

To determine the degradation efficiency of the catalysts, the kinetic analysis was carried out with methylene blue (MB) and fipronil pesticide. About 20 mL (5 ppm) of MB dye solution having 0.1 g L^−1^ of the as-prepared catalysts was allowed to stir firstly in dark for 30 min to attain adsorption equilibrium and then in visible light for 80 min for photodegradation. To compare the effectiveness of the catalyst, a similar degradation experiment was performed with all the composites as well as bare MoS_2_ and MoSe_2_. The degradation efficiency, rate constants, and synergy factors of as-prepared catalysts were analyzed. The photocatalysts show much higher degradation efficiency (Fig. 5) than commercial TiO_2_ nanopowder (Degussa, P25). It was observed that the photocatalytic efficacy of all the prepared composites was higher than the individual MoS_2_ and MoSe_2_ catalysts (Fig. 5a). The synergy attained from the combination of MoS_2_ and MoSe_2_ photocatalytic system can be determined with help of synergy factor (*R*) as follows:3
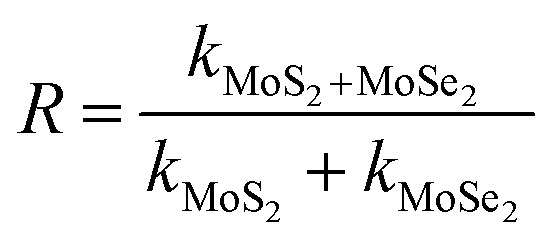
where *k*_MoS_2_/MoSe_2__, *k*_MoS_2__, and *k*_MoSe_2__ in the eqn (3) are the photodegradation rate constants of MoS_2_/MoSe_2_ composite, pure MoS_2_, and pure MoSe_2_ respectively.^35^ The synergy factors for different MSMSe composites calculated from the above equation are 0.98, 1.34, and 1.73 for MSMSe (1 : 1), MSMSe (3 : 1), and MSMSe (1 : 3) photocatalysts respectively. Although the photocatalytic activity of all the composites was high, however the best degradation efficiency (∼94%) was achieved by MSMSe (1 : 3) catalyst with a high rate constant of 0.033 min^−1^ and strong synergistic effect (*R* = 1.73). This high efficiency is may be due to the higher surface area (confirmed from BET surface area analysis) and lower recombination rate (confirmed from PL studies) of MSMSe (1 : 3) nanocomposite which facilitates the higher adsorption on the catalyst surface and degradation process.

To draw the correlation among different light sources, a similar trial was led in UV light and sunlight with MSMSe (1 : 3) as a catalyst and summarized in Table 2. The efficiency of degradation follows the order as sunlight > UV light > visible light which confirms that only the small amount of catalyst is highly effective and can be used directly under sunlight irradiation.

**Table 2:** Photocatalytic activities of MSMSe (1 : 3) composite for MB degradation under different light sources

Light source	Percent degradation (%)	Rate constant (min^−1^)
Ultraviolet	96	0.0338
Visible	94	0.0332
Sunlight	97	0.0345

Likewise, to make the differentiation that the system of photocatalysis is direct or indirect, the photodegradation of colorless pesticide fipronil was conducted. For this purpose, about 10 mL (600 ppm) of fipronil solution and 2 mg of the prepared catalysts were allowed to stir in the dark for 30 min and then in visible light up to 80 min for photodegradation. The studies were also conducted in similar conditions without adding any catalyst to the solution, which shows only 8% degradation in light. Whereas after adding the catalyst, the efficiency of degradation increases by up to 80% (Fig. 5b) with a high rate constant of 0.0168 min^−1^ by MSMSe (1 : 3) composite, which confirms that the photodegradation was indirect. Table 3 shows the comparison of degradation efficiency of as-prepared photocatalysts with similar kind of catalysts reported in the literature, which shows that the as-prepared catalysts are one of the efficient photocatalysts with a high value of degradation efficiency and rate constant in visible light.

**Table 3:** Photocatalytic activity comparison of as-prepared catalysts with literature

Photocatalyst	Pollutant	Catalyst concentration (g L^−1^)	Degradation (%)	Degradation rate (min^−1^)	Reaction time (min)	Ref.
Crystalline MoSe_2_	Rhodamine B	2	90	0.019	120	36
MoSe_2_/SrTiO_3_	Methyl orange	0.2	99.46	—	70	37
TiO_2_/MoS_2_/TiO_2_	Methyl orange	—	89.86	0.017	150	38
SnO_2_/MoS_2_	Methylene blue	—	58.5	0.022	120	39
Fe_2_O_3_/MoS_2_	Methylene blue	0.1	84	—	120	40
MoSe_2_/pg-C_3_N_4_	Tetracycline	0.5	92	0.0281	80	41
TiO_2_ (Degussa P25)	Methylene blue	0.1	25	0.0026	80	Present study
MoS_2_	Methylene blue	0.1	68	0.0088	80	Present study
MoSe_2_	Methylene blue	0.1	69.4	0.0103	80	Present study
MoS_2_/MoSe_2_ (1 : 1)	Methylene blue	0.1	82.1	0.0188	80	Present study
MoS_2_/MoSe_2_ (3 : 1)	Methylene blue	0.1	91.3	0.0257	80	Present study
MoS_2_/MoSe_2_ (1 : 3)	Methylene blue	0.1	94	0.0332	80	Present study

#### 3.2.2 Catalyst concentration effect

**Fig. 6:**
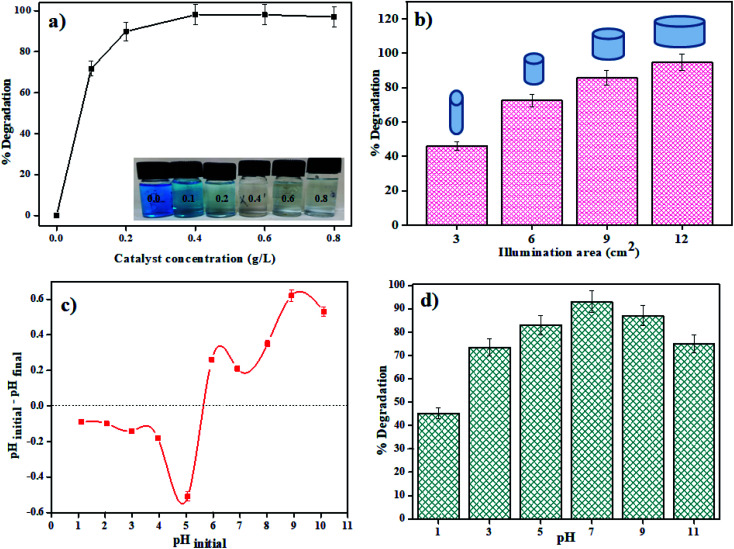
Plots showing effect of (a) catalyst concentration, (b) illumination area, (c) point zero charge, and (d) pH of MB dye solution on photodegradation by the MSMSe (1 : 3) nanocomposite.

The optimum concentration of catalysts was determined by varying the catalyst dose and carrying out the photodegradation of MB dye in visible light. For this, the concentration of MSMSe (1 : 3) catalyst was varied from 0.1 to 0.8 g L^−1^ in 20 mL of MB dye solution (5 ppm). From Fig. 6a, we can see that as the concentration rises from 0.1 to 0.4 g L^−1^ there is a significant increase in the degradation efficacy after 80 min of photodegradation. However, after this, if the concentration was further increased (0.4 to 0.8 g L^−1^), there was no noticeable increase in the efficiency. The saturation in degradation efficiency may be due to the increase in light scattering due to a highly opaque solution.

#### 3.2.3 Illumination area effect

The role of the total effective illumination area was as well investigated in visible light using MSMSe (1 : 3) as a photocatalyst. About 1 mg of the catalyst was stirred in 20 mL of MB dye (5 ppm) solution in the visible light for 80 min. The diameter of the reaction surface was varied by using vessels of different sizes keeping all other parameters same throughout the experiment. The distance between the light source and the upper layer of the solution was kept constant (10 cm) while using different vessels. There is an increase in the degradation efficacy with an increase in the area of the exposed reaction mixture to light irradiation (Fig. 6b). The greater the exposed area of the reaction mixture more will be the absorption of light which further facilitates the photocatalytic degradation of the pollutants and thus showing an increase in degradation efficiency.

#### 3.2.4 Solution pH effect

The capacity of the photocatalyst to absorb contaminations on its surface is significantly impacted by the pH of the solution, which further influences the efficiency of degradation. To study the influence of pH on degradation efficacy, point zero charge (pzc) of the catalyst surface was measured. The studies were carried out by varying the pH of MB dye solution using 0.1 N HCl or NaOH solution. In 20 mL of the MB dye solution, 1 mg of MSMSe (1 : 3) photocatalyst was added and the pH value before and after the treatment was noted. The results of the study show that the pzc of the catalyst was around pH 5.6 (Fig. 6c). This shows that there was no significant degradation occurs in acidic pH (below pH 5.6). This is because the adsorption of cationic adsorbents (MB) is supported if pH > pzc where the catalyst species exist as M–O^−^ (M is the metal).^42^ As MB dye is cationic so it will absorb more productively on the catalyst surface above pH 5.6. So, the maximum degradation efficiency was observed at pH 7 as shown in Fig. 6d. However, at higher basic pH (above pH 9) the degradation efficacy decreases as precipitates of metal hydroxides may get accumulated on the catalyst surface which deactivates the catalyst.

#### 3.2.5 Real wastewater treatment

**Fig. 7:**
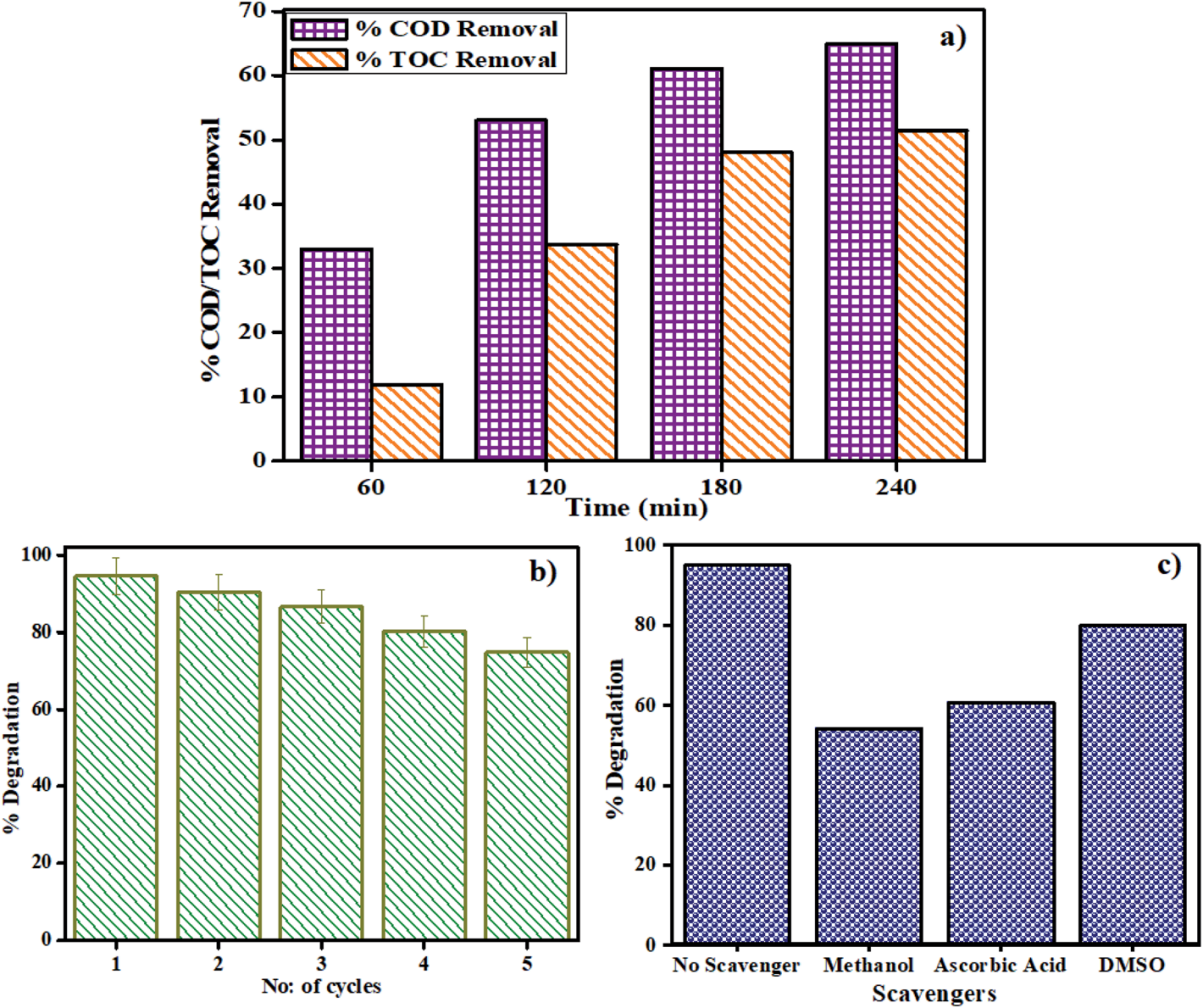
(a) Percent COD and TOC removal in real textile wastewater, (b) reusability, and (c) scavenger studies in MB dye solution with MSMSe (1 : 3) photocatalyst.

Real industrial wastewater was utilized to measure the viability of the as-synthesized catalyst dependent on the ideal conditions. The amount of organic matter in the wastewater was determined with the help of COD (Chemical Oxygen Demand) and TOC (Total Organic Carbon) techniques. The COD value of industrial wastewater without any treatment was found to be 1950 mg L^−1^, which shows that the amount of organic matter in the wastewater is high. So photodegradation of this wastewater by MSMSe (1 : 3) catalyst under optimum conditions (4 mg catalyst in 50 mL wastewater) was carried out and the COD and TOC removal percentage were analyzed at regular time intervals as shown in Fig. 7a. After 240 min of photodegradation, the COD and TOC removal percentage were increased to 65% and 51.5% respectively. As we have used raw industrial wastewater so we followed higher degradation time for better mineralization of pollutants. The increase in removal percentage with time shows that the organic matter present in the wastewater is mineralizing to relatively simpler compounds. Moreover, the average oxidation state (AOS) and carbon oxidation state (COS) values were calculated from eqn (4) and (5) below in order to determine the biodegradability variation of real industrial wastewater:^43^

4 AOS = 4 − 1.5[COD/TOC]

5 COS = 4 − 1.5[COD/TOC_i_]

The values of AOS and COS variables have a range of +4 to −4 for the most oxidized form *i.e.* CO_2_ and the most reduced form *i.e.* methane.^44^ The initial AOS value of real industrial wastewater without any treatment was 1.73 which increased up to 2.37 and 3.2 for AOS and COS respectively after the photocatalytic degradation. The results show that the biodegradability of real wastewater has been increased in the presence of MSMSe (1 : 3) photocatalyst and the catalyst is effective for the treatment of effluents than the physico-chemical treatment performed by the industries.

#### 3.2.6 Reusable photocatalyst

One of the major challenges in the photodegradation method of pollutant removal is that the catalyst cannot be reused, as dye molecules might react with the catalyst or the separation of the catalyst from the solution is very tedious. The as-prepared catalysts are not only easily separable by mere centrifugation but can also be reused with great effectiveness. The reproducibility of the catalyst was investigated by stirring MSMSe (1 : 3) catalyst in 20 mL of MB dye (5 ppm) under visible light for 80 min and after the degradation; the catalyst was separated by centrifugation with distilled water washing. Then the regained catalyst was used again and the process was repeated up to 5 runs as shown in Fig. 7b. The results show that after 5 runs the degradation efficacy of the catalyst was still very high (∼75%), which confirms that the catalyst is highly reusable. To confirm the reusable nature of the catalyst the XRD pattern, FESEM image, PL spectra, and BET isotherms of MSMSe (1 : 3) nanocomposite before and after 5 cycles of photocatalytic degradation is shown in Fig. SI 4 (ESI†). The majority of diffraction peaks of the catalyst are still present even after 5 cycles of degradation (Fig. SI 4a†) verifies that the crystal structure of MSMSe (1 : 3) nanocomposite is not disturbed after the reaction which is due to the high photocorrosion resistance of MoSe_2_. The peaks after the degradation are still sharp which shows that the crystallinity of the catalyst was maintained even after many degradation cycles. Also, the morphology of the catalyst remains intact as shown by the FESEM image of MSMSe (1 : 3) composite after 5 cycles of degradation (Fig. SI 4b†). Fig. SI 4c† shows the PL spectra before and after degradation up to 5 cycles. There is a slight increase in the PL intensity after degradation, which may be due to the deactivation of some active sites. The surface area of the MSMSe (1 : 3) photocatalyst before degradation was 60 m^2^ g^−1^ which decreases to 48 m^2^ g^−1^ after 5 cycles of degradation which is still high and this confirms that the pollutants were not permanently adsorbed over the surface of the catalyst and thus show high degradation efficiency even after several degradation cycles. The BET isotherms of the MSMSe (1 : 3) catalyst before and after degradation cycles were shown in Fig. SI 4d.† All these studies confirm that the catalyst is highly stable and reusable with great degradation efficiency.

#### 3.2.7 Plausible photocatalysis mechanism

**Scheme 2:**
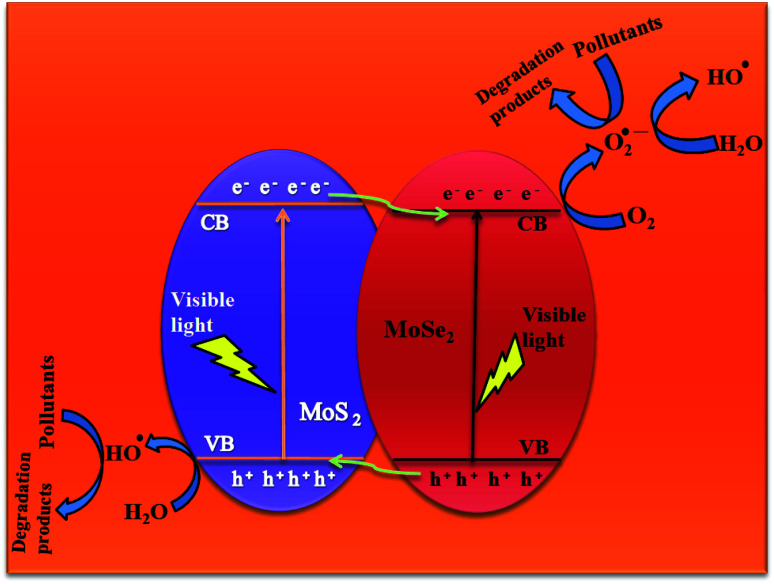
Plausible photocatalytic degradation mechanism.

To determine the major species governing photocatalytic degradation and mechanism of photocatalysis, scavenger studies were carried out with different scavengers like methanol, DMSO, and ascorbic acid for trapping holes (h^+^), electrons (e^−^), and superoxide radicals (O_2_^−^), respectively.^45^ The degradation efficiency was carried out by comparing the activity of MSMSe (1 : 3) catalyst in the absence and presence of different scavengers as shown in Fig. 7c. The results demonstrate that the catalytic efficiency was most affected in the case of methanol as the degradation efficiency decreases to 54% in presence of methanol which means holes participate maximum in the degradation process. However, the degradation efficacy was significantly decreased in the case of ascorbic acid (60.8%), which confirms the role of superoxide radicals in degradation. This shows that the degradation of dye was influenced primarily by the holes and superoxide radicals. Therefore, based on trapping experiments the plausible mechanism of photocatalysis is shown in Scheme 2. As the bandgap energies of MoS_2_ and MoSe_2_ are tunable and both are active in the visible light, so as it absorbs light energy, the electrons in its valence band get excited to their conduction band level leaving the holes behind. Due to the formation of heterojunction between the two semiconductors, these electron–holes do not recombine and thus a charge separation occurs. Now these electrons in the CB react with the molecular oxygen forming the superoxide radicals which can either further react with a water molecule to form hydroxyl radicals or can directly degrade the pollutants. On the other hand, the holes created in the VB can react with water to form hydroxyl radicals which further cause the degradation of pollutants to either CO_2_ and water or relatively simpler products. The probable reaction steps ((6)–(11)) are:

6 MoS_2_/MoSe_2_ + *hv* → e^−^ + h^+^

7 H_2_O + h^+^ → H_2_O^+^ → OH^−^ + h^+^

8 O_2_ + e^−^ → O_2_^−^˙ + h^+^

9 H_2_O^+^ → OH^−^ + h^+^

10 OH^−^ + h^+^ → OH˙

11 OH˙/O_2_^−^˙ + pollutants → degraded products

### 3.3 Electrocatalytic studies

#### 3.3.1 HER activity

**Fig. 8:**
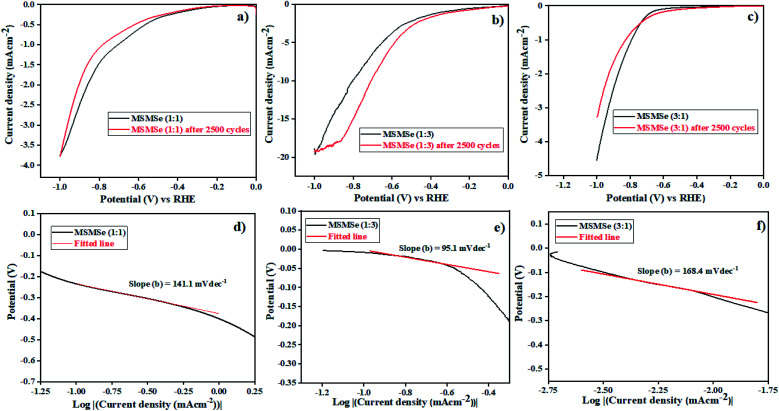
LSV plots of (a) MSMSe (1 : 1), (b) MSMSe (1 : 3) and (c) MSMSe (3 : 1) composites in acidic medium and Tafel plots of (d) MSMSe (1 : 1), (e) MSMSe (1 : 3) and (f) MSMSe (3 : 1) composites.

The electrochemical activities of as-synthesized nanocomposites were analyzed in 0.5 M H_2_SO_4_. The polarization curves in acidic medium obtained for as-prepared composites are shown by linear sweep voltammetry (LSV) plots in Fig. 8(a–c). These plots show a maximum current density of −3.76 mA cm^−2^ and −4.52 mA cm^−2^ at a potential of −1 V for MSMSe (1 : 1) and MSMSe (3 : 1) composites respectively. However, the maximum current density of −19.4 mA cm^−2^ has been obtained for MSMSe (1 : 3) catalyst at −1 V potential (Fig. 8c) in the acidic medium. The MSMSe (1 : 3) composite exhibits high surface area and the presence of micropores and mesopores with enlarged pore volume, which increases the active surface characteristics of the composite and hence results in enhanced HER performance.^22^ The MSMSe composites show high current density even with very low mass loading which confirms high efficiency of the prepared catalysts.

The performance and efficiency of the electrocatalyst were most prominently defined by its stability which is required in the present time. The stability of MSMSe nanocomposites was determined by the cyclic voltammetry (CV) technique performed for 2500 cycles at a scan rate of 100 mV s^−1^ in 0.2 to 0.6 V voltage window. The polarization curves obtained after the 2500 CV cycles of the composites show that for MSMSe (1 : 1) and MSMSe (1 : 3) composites, there is a negligible loss in current density as the maximum current density of −3.74 mA cm^−2^ and −19.32 mA cm^−2^ was observed even after 2500 CV cycles as shown in Fig. 8a and c. The combination of MoS_2_ and MoSe_2_ gives rise to a synergistic effect which provides an open porous space to facilitate the charge transfer within the interlaced arrays so we observe high stability in current density. Moreover, the higher stability of the as-synthesized composites is attributed to their high acidic corrosion resistance under continuous CV cycling (under continuous oxidation/reduction process). However, in the case of MSMSe (3 : 1) composite, there is a slight decrease of current density to −3.28 mA cm^−2^ after 2500 CV cycles. This is due to the higher charge transfer resistance in MoS_2_ as the metallic character of MoS_2_ is lower as compared to MoSe_2_ (as the metallic nature of S is less).^46^

To determine the mechanism responsible for the HER and electrocatalytic performance, Tafel slope is one of the most important analysis. The following steps determine the probable mechanism responsible for HER performance:^47^

12 Volmer: H_3_O^+^ + M(e^−^) → MH_ads_ + H_2_O

13 Heyrovsky: H_3_O^+^ + MH_ads_ + M(e^−^) → 2M + H_2_O + H_2_

14 Tafel: 2MH_ads_ → 2M + H_2_

Here the M determines the species active sites and MH_ads_ denote the hydrogen adsorbed intermediate. In the Volmer reaction (eqn (12)) on the catalyst surface a proton gets attached and then through Heyrovsky and Tafel reactions (eqn (13) and (14)) the produced hydrogen leaves the surface of catalyst. If the Tafel slope of the reaction is ∼120 mV dec^−1^ then the reaction proceeds through Volmer–Heyrovsky pathway whereas if the value is ∼30 mV dec^−1^ then the reaction follows Volmer–Tafel pathway. The reaction rate will be higher if the value of Tafel slope is smaller with respect to the given potential.^48^ The Tafel slopes of the MSMSe composites are shown in the insets of their LSV plots (Fig. 8(d–f)). The value of Tafel slope for MSMSe (1 : 1), MSMSe (1 : 3) and MSMSe (3 : 1) are 141.1, 95.1 and 168.4 mV dec^−1^ respectively in acidic medium. This shows that in an acidic electrolyte, the rate-determining step (RDS) for HER is the Volmer–Heyrovsky reaction. The lower value of Tafel slope for MSMSe (1 : 3) nanocomposite shows the faster charge transfer kinetics in the heterostructure. The edge sites of MoS_2_/MoSe_2_ are the active species in an acidic medium for HER. So the lower Tafel slope value in MSMSe (1 : 3) shows a higher number of active edge sites in the composite which may be due to its higher surface area. The values predict that adsorption of H^+^ ion is fast but the desorption process is slow due to higher surface area and pore size. The active sites get more exposed due to the abundant interfaces in MoS_2_/MoSe_2_ heterostructure for the electrochemical reaction. The high stability of MSMSe nanocomposites and faster charge transfer kinetics on the active edge sites of heterostructure makes them potential catalysts for HER.

#### 3.3.2 Electric double layer capacitance (EDLC) or *C*_dl_

**Fig. 9:**
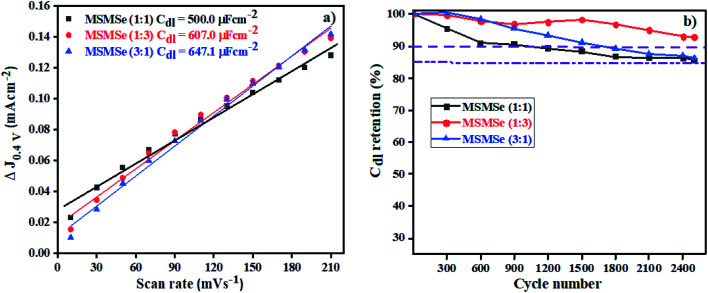
(a) EDLC plot and (b) capacitance retention with a number of cycles for MSMSe composites.

The storage capacity of the as-synthesized nanocomposites has been determined from the CV plots performed at various scan rates of 10–210 mV s^−1^ in the voltage window of 0.2 to 0.6 V as shown in Fig. SI 5(a–c).† The rectangular shape of CV plots suggests that the reactions are reversible and occur without faradic reaction and attributes to the EDLC (*C*_dl_) storage capability. By plotting the scan rate *vs.* current density variation (Δ*J* = *J*_cathodic_ − *J*_anodic_ at 0.4 V) the calculated *C*_dl_ values (Fig. 9a) comes out to be 500.0, 607.0, and 647.1 μF cm^−2^ for MSMSe (1 : 1), MSMSe (1 : 3) and MSMSe (3 : 1) respectively suggesting that the MoS_2_ and MoSe_2_ heterojunction leads to the higher energy storage performance due to the presence of more exposed active sites in the heterostructure. From the CV plots, one can observe that even when the CV was performed at a lower speed (10 mV s^−1^) there was no change observed in the shape of CV plots, only the voltage/area was enhanced. It also shows that the prepared composites show higher electrochemical active surface area (ECAS) and results in the enhancement of their electrochemical performance. The stability and charge storage ability is the vital parameter for electrode fabrication for electrochemical capacitors. The capacitance retention was determined *via* CV analysis at a fixed scan rate of 100 mV s^−1^ for 2500 CV cycles in an acidic medium in a 0.2 to 0.6 V voltage range for MSMSe composites (Fig. SI 5(d–f)†). It can be observed from the figures that the shape of CV plots was retained even after 2500 cycles. The only change was observed in the first CV cycle after which all the CV cycles remain the same. The enhanced retention of capacitance in acidic medium was confirmed by plotting the % capacitance retention with respect to CV cycle number (Fig. 9b), which shows that MSMSe (1 : 3) has the highest capacitance retention of ∼94% for 2500 CV cycles compared with bare MoS_2_ in literature showing stability up to only 1000 cycles.^49^ All above results confirm that the as-prepared MSMSe composites are the potential electrode material for capacitor applications.

## 4. Conclusion

In summary, the 2D/2D heterojunction of MoS_2_/MoSe_2_ with different weight ratios was successfully prepared by the facile microwave technique which is less time taking and cost-effective. The prepared materials show high photocatalytic and electrocatalytic performance. Compared with the pure MoS_2_ and MoSe_2_, the photocatalytic efficiency of the composites was highly enhanced towards MB dye and fipronil pesticide degradation. Due to their similar energy levels, the heterojunction formed between the two, assist the separation of charges which results in the better degradation activity of the composites. The degradation efficiency was remarkably affected by varying the amount of MoS_2_ and MoSe_2_ in the composite. The MSMSe (1 : 3) composite shows the best catalytic activity due to its higher surface area and lower recombination rate of charges as compared to other composites. Moreover, the pH of the solution, the amount of catalyst, and the exposed area has a high impact on the degradation efficiency of the present catalysts. The high removal of COD and TOC in the real wastewater confirms the as-prepared catalysts can be used in the physico-chemical treatment of real wastewater. Also, the recovery and reuse of the catalyst make it suitable for long-term application. In the acidic medium, the synthesized nanocomposites are highly efficient electrocatalysts for HER activity with a stable current density. The EDLC studies show that the MSMSe nanocomposites are potential electrode material with high capacitance retention for energy storage devices. The studies in this work can give a genuine guide for the fast and easy synthesis of highly efficient; visible-light based stable catalysts for photocatalytic wastewater treatment and electrocatalytic hydrogen production.

## Conflicts of interest

The authors have no conflict of interest in the publication of this manuscript.

## Supplementary Material

RA-011-D1RA01760H-s001
