# Supplementary material for: Tuning the photocatalytic/electrocatalytic properties of MoS2/MoSe2 heterostructures by varying the weight ratios for enhanced wastewater treatment and hydrogen production
Source: RSC Adv. 2021 Jun 28;11(37):22585–97. doi: 10.1039/d1ra01760h (PMC9034378; doi:10.1039/d1ra01760h)
Supplement: RA-011-D1RA01760H-s001 [file RA-011-D1RA01760H-s001.pdf]

Electronic Supplementary Material (ESI) for RSC Advances.
This journal is © The Royal Society of Chemistry 2021

Supporting Information

Tuning the Photocatalytic/Electrocatalytic Properties of $MoS_2/MoSe_2$ Heterostructures by Varying the Weight Ratios for Enhanced Wastewater Treatment and Hydrogen Production

Divya Monga and Soumen Basu*

School of Chemistry and Biochemistry, Affiliate Faculty—TIET-Virginia Tech Center of Excellence in Emerging Materials, Thapar Institute of Engineering and Technology, Patiala-147004, India

Materials used

Analytical grade reagents and double distilled water for the preparation of solutions was used throughout the experiment. Methylene blue dye [$C_{16}H_{18}ClN_3S$, λ_{max} = 664nm] was acquired from Spectrochem Pvt. Ltd. India. Ammonium molybdate tetrahydrate (98%) [$(NH_4)_6Mo_7O_{24}.4H_2O$], selenous acid (H_2SeO_3), ferrous ammonium sulphate (FAS) and potassium dichromate ($K_2Cr_2O_7$) was purchased from Loba Chemie Pvt. Ltd. India. Fipronil 5% SC was acquired from Bayer Crop Science Ltd. India. Real waste water was procured from textile industry, Ludhiana, India. Silver sulphate (Ag_2SO_4), mercuric sulphate ($HgSO_4$) and ferroin indicator was purchased from Rankem.

Catalyst Characterization

The X-ray diffraction analysis (XRD) of as-prepared catalysts was performed by using PANalytical X-ray diffractometer (Ni filtered Cu Kα radiations with λ = 0.1504 nm at 45 kV) with a scan range of 10–90° and scan rate of 2° per min. To analyze the surface area of the

compounds, BET surface area analyzer of Microtrac Belsorp Mini-II (Bel, Japan, Inc) was used, at −196 °C. The samples were first pretreated at 523 K to get a clean surface for adsorption isotherm. The pore size distribution was calculated from N_2 adsorption-desorption isotherms by using Barrett– Joyner–Halenda (BJH) model. The photodegradation was performed in the UV photoreactor (100 W Hg lamp emitting ultraviolet light (365 nm)). A UV-2600 spectrophotometer (AVANTES) was used to record the UV–visible diffuse reflectance spectra (DRS) of prepared nanocomposites from 180 to 1100 nm. The reference material used was Barium sulphate ($BaSO_4$). The Field emission scanning electron microscope (FESEM) and energy dispersive X-ray spectroscopy (EDS) of MIRA3 TESCAN (accelerating voltage of 15kV) and high resolution transmission electron microscope (HRTEM) of Thermofisher of model Talos F200 S has been used for the structural studies of the as prepared catalyst. Photoluminescence studies were carried out on fluorescence spectrophotometer (PerkinElmer LS-55, USA) using an excitation wavelength of 320 nm. The oxidation state and chemical composition of the as prepared catalyst was analyzed by using, XPS spectrometer of Omicron ESCA + (Al Kα radiation having hν = 1486.7 eV). The TOC analyzer of (multi N/C 2100) Analytik Jena AG Corporation, Germany were used for TOC measurments.

Photocatalytic studies

All the experiments were performed in triplicates. To determine the photocatalytic activity of the catalysts, 1 mg of catalyst was put into the MB dye solution (20 mL of 5 ppm) and 2 mg of the catalysts into the fipronil solution (10 mL of 600 ppm). The adsorption-desorption equilibrium was established by stirring the solution for 30 min in a completely dark environment. Then the reaction solution was illuminated by UV lamp having a λ_{max} of 365 nm (100 W Hg lamp) having

flux density in the range of 46-48 W/m^2, visible light (65W CFL lamp, Phillips, k > 400 nm with intensity of 125 W/m^2) and sunlight (at TIET, Patiala, India during 17/06/2019 to 24/06/2019 between 10am to 3 pm with an average intensity of 426 W/m^2) upto 80 min. The powder catalyst was removed from the solution by centrifugation. UV-Vis spectrophotometer (Champion) was employed to investigate the absorbance spectra of MB and fipronil. The photodegradation efficiency was determined using the relation:

$$\text{Degradation efficiency} = \{(C_0 - C) / C_0\} \times 100 = \{(A_0 - A)/ A_0\} \times 100 \quad (1)$$

where C_0, C: concentration of MB dye solution at 0 and t time, respectively and A_0 and A: absorbance of MB solution at 0 and t time, respectively.

COD (chemical oxygen demand) and TOC studies were also done to study the degradation of raw industrial waste water. The COD values were determined using the titrimetric method. TOC was used to study the mineralization of real waste water. The COD and TOC measurements were done before and after 240 min of photocatalytic treatment of polluted water under visible light irradiation using 0.4g/L of MSMSe (1:3) photocatalyst.

Percent COD and TOC were calculated using:

$$\%COD = \{(COD_0 - COD_t)/ COD_0\} \times 100 \quad (2)$$

where COD_0, COD_t are the COD of real waste water at time ‘0’ and‘t’ respectively.

$$\%TOC = \{(TOC_0 - TOC_t)/ TOC_0\} \times 100 \quad (3)$$

where TOC_0, TOC_t are the TOC of real waste water at time ‘0’ and‘t’ respectively.

Electrocatalytic studies

The as-prepared samples were investigated for their electrocatalytic activity test using Bio-logic EC Lab SP300 standard setup. Out of the three electrodes in the system; platinum was used as the counter electrode, whereas the working electrode was prepared manually. For fabrication of working electrode, 1mg of the catalyst was mixed with 250 µL and the solution was sonicated for 25 min to achieve uniform dispersion of particles. The top of glassy carbon electrode (GCE), which has the surface area of 0.070 cm^2, was drop-casted by 20 µL of the above dispersed solution. A single drop of Nafion 117 solution (Sigma Aldrich) from 10 µLwas put onto the GCE top and was dried overnight. The measurements were done using reversible hydrogen electrode (RHE) as the reference electrode in the 0.5M H_2SO_4 electrolyte solution.

EDS and Color mapping

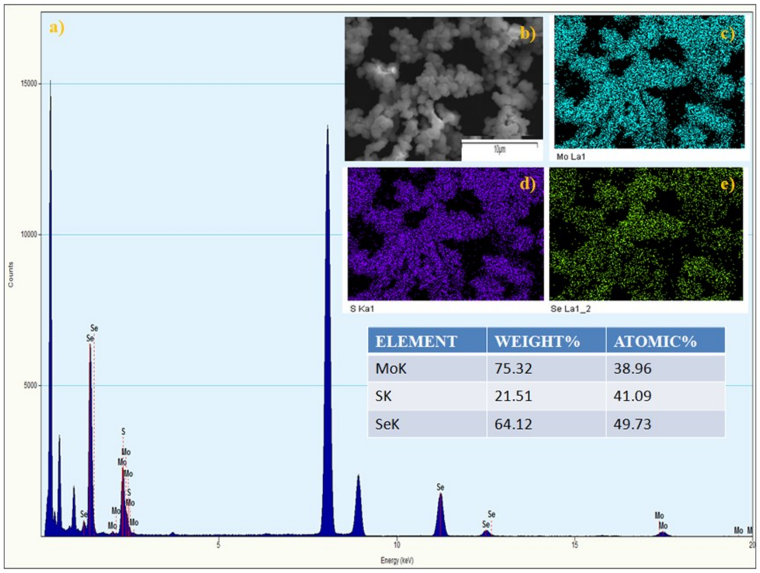

Fig. SI1 (a) EDS spectra and **(b – e)** color mapping of MSMSe (1:3) photocatalyst.

DRS and Tauc plot

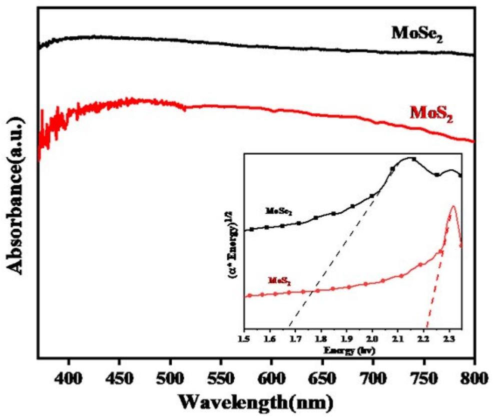

Fig. SI2 DRS absorption spectra and Tauc plots (inset) of MoS_2 and $MoSe_2$ catalysts.

Surface area analysis

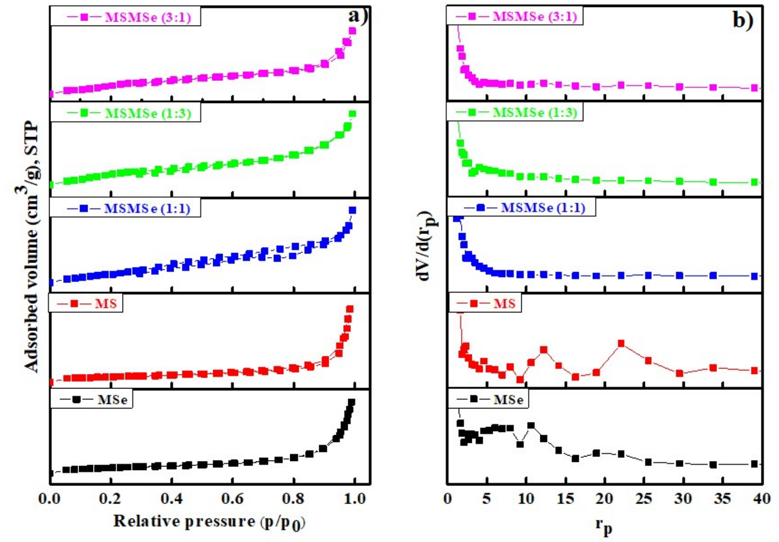

SI3. (a) Adsorption-desorption isotherms and (b) BJH pore size distribution curves for MSMSe nanocomposites.

Reusability studies

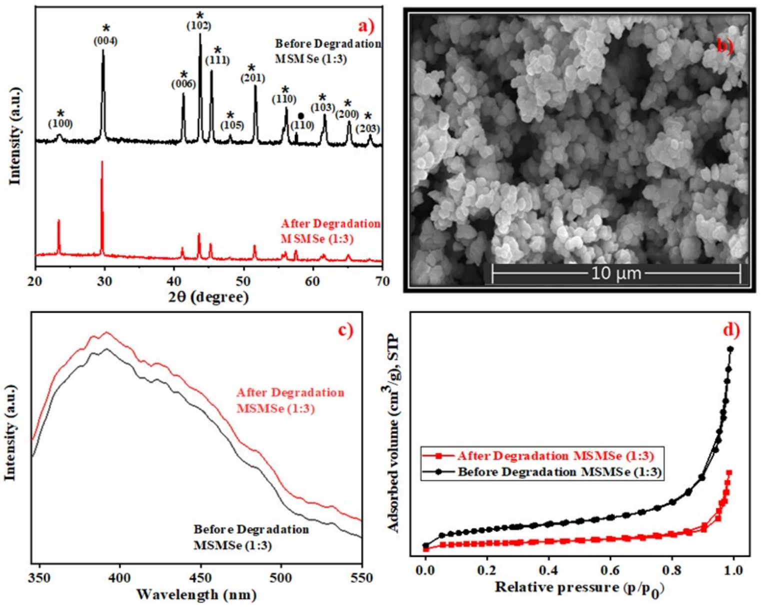

Fig. SI4. (a) The XRD spectra, **(b)** FESEM image, **(c)** photoluminescence spectra, and BET adsorption isotherms of MSMSe (1:3) catalyst before and after 5 cycles of photocatalytic degradation of MB.

Electric Double Layer Capacitance (EDLC) or C_{dl}

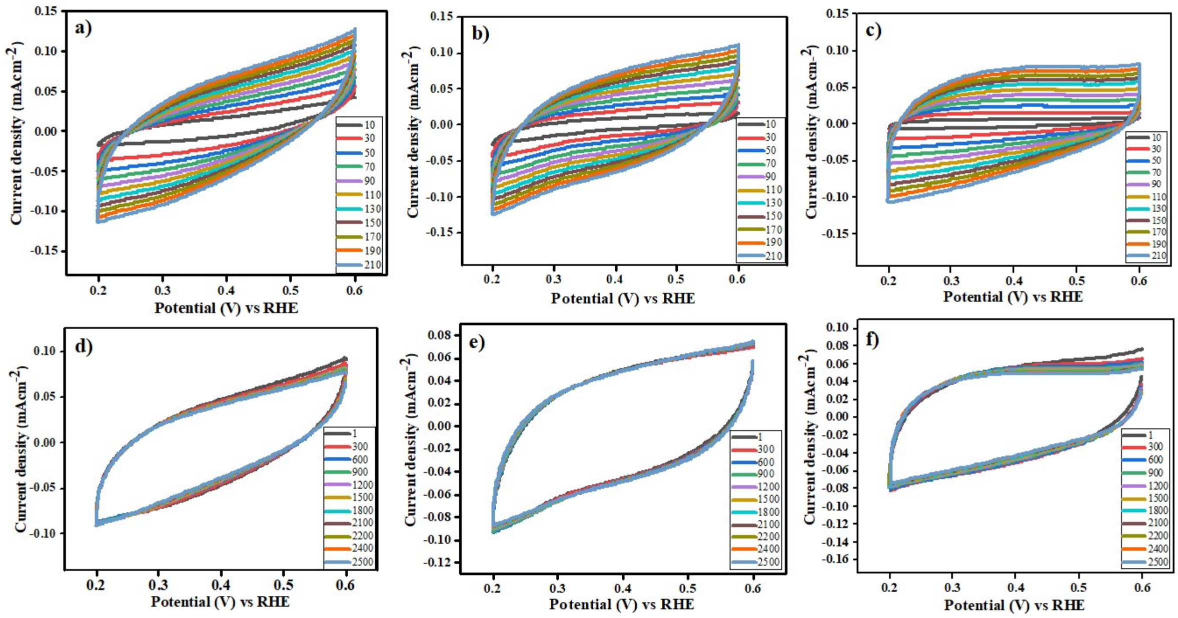

Fig. SI5 CV plots of (a) MSMSe (1:1), (b) MSMSe (1:3), and (c) MSMSe (3:1) at multiple scan rates and CV plots for 2500 cycles of (d) MSMSe (1:1), (e) MSMSe (1:3), and (f) MSMSe (3:1).
